# Efficacy of Ultrasound Versus Landmark-Guided Steroid Injections for Carpal Tunnel Syndrome: A Randomized Double-Blind Trial

**DOI:** 10.5152/ArchRheumatol.2026.25100

**Published:** 2026-01-16

**Authors:** Nur Topcu-Altın, Muhammet Hüseyin Sarı, Meral Bilgilisoy-Filiz, Şebnem Koldaş- Doğan, Hasan Fatih Çay, Naciye Füsun Toraman

**Affiliations:** Physical Medicine and Rehabilitation Clinic, Antalya Training and Research Hospital, Antalya, Türkiye

**Keywords:** Carpal tunnel syndrome, corticosteroid injection, landmark-guided, midterm outcomes, ultrasound-guided

## Abstract

**Background/Aims:**

This study compared the mid-term effectiveness of ultrasound-guided (USG) vs. landmark (LM)-guided corticosteroid injections in patients with moderate carpal tunnel syndrome (CTS), evaluating symptom severity, functional status, sonographic and electrophysiological parameters, and complications over 6 months.

**Materials and Methods:**

A prospective, randomized, double-blind trial was conducted on 168 wrists (84 participants) with bilateral moderate CTS. Participants were divided into LM-guided and USG injection groups. Primary outcomes included the Boston Carpal Tunnel Syndrome Questionnaire (BCTQ) Symptom Severity Scale (SSS) and Functional Status Scale (FSS). Secondary outcomes were grip strength (GS), median nerve cross-sectional area (MNSA), median nerve flattening ratio (MNFR), and electrophysiological parameters. Assessments were performed at baseline, 1 month, and 6 months post injection.

**Results:**

Both groups showed significant improvements in BCTQ-SSS, BCTQ-FSS, GS, MNSA, MNFR, and electrophysiological parameters at 1 and 6 months (*P* < .05). No significant differences were observed between the 2 techniques in efficacy. However, hypopigmentation occurred more frequently in the LM group (11% vs. 3%, *P* = .04). No severe complications were reported.

**Conclusion:**

The USG and LM-guided corticosteroid injections are equally effective for moderate CTS over 6 months. While USG may reduce minor complications like hypopigmentation, both methods are safe and viable options when performed by experienced clinicians.

**Main Points**

- Both ultrasound-guided (USG) and landmark (LM)-guided corticosteroid injections significantly improve symptoms, function, and electrophysiological findings in moderate carpal tunnel syndrome (CTS).
- There is no significant difference in clinical efficacy between USG and LM-guided injections at 6 months.
- Ultrasound-guided injections are associated with fewer minor complications such as hypopigmentation compared to LM-guided injections.
- Both injection techniques are safe when performed by experienced clinicians and can be used as viable treatment options for moderate CTS.
- A 6-month follow-up provides evidence for the mid-term effectiveness and safety of both techniques.

## Introduction

Carpal tunnel syndrome (CTS) is a neuropathy of the median nerve at the wrist level caused by compression of the flexor retinaculum. Compression of the median nerve results in nerve ischemia, impaired nerve conduction, and ultimately nerve damage. Increased pressure within the carpal tunnel is the primary pathophysiologic mechanism implicated in etiology.^1^^,^^2^ The CTS is the most common form of peripheral entrapment neuropathy. It accounts for approximately 90% of all entrapment neuropathies. Pressure within the carpal tunnel is observed to be at its lowest level when the wrist is at neutral or slightly flexed. Although most cases are idiopathic, concomitant conditions such as trauma, occupational exposure, diabetes, hypothyroidism, obesity, pregnancy, vascular lesions, advanced age, osteoarthritis, or rheumatoid arthritis also increase the incidence of CTS. The most common symptoms of CTS are numbness, tingling, electric shock, and burning sensations radiating from the wrist to the distribution area of the median nerve.^3^^-^^5^

The diagnosis of CTS is based on a detailed clinical history and clinical assessment. However, the most reliable method for both diagnosis and staging is electrodiagnostic studies (EDx). The EDx has 84% specificity and 99% sensitivity to confirm the diagnosis of CTS.^6^

Treatment options for CTS include splinting, exercise, nonsteroidal anti-inflammatory drugs, corticosteroid injections, and various physical therapy modalities. Surgical decompression may be considered for patients whose symptoms persist despite conservative treatment.^1^^,^^6^

Clinicians widely use corticosteroid injections as a highly effective and safe treatment option for mild to moderate CTS.^7^ A single injection can provide symptom relief for approximately 50% of patients for up to a year without requiring additional treatment. It is believed that corticosteroid injections reduce inflammation and edema within the carpal tunnel, thereby lowering pressure and alleviating mechanical compression on the median nerve, facilitating recovery.^8^^,^^9^

In clinical practice, corticosteroid injections are commonly administered using blind techniques by palpating superficial anatomical landmarks (LM).^10^Although major complications are rare, there is a risk of injury to the median nerve, tendons, and vascular structures. Additionally, if the corticosteroid injected blindly does not adequately reach the carpal tunnel, its effectiveness may be reduced.^11^ Consequently, ultrasonography (USG) in clinical practice is steadily increasing.

Most studies have demonstrated that ultrasound-guided corticosteroid injections enhance effectiveness and safety. However, when the literature was reviewed, comparisons between these 2 techniques primarily focused on short-term outcomes, while mid- and long-term results remain insufficiently explored.^12^^-^^14^ To address this gap, this study aimed to compare the mid-term effectiveness of USG vs. LM-guided corticosteroid injections on symptoms, functional status, and electrophysiological and ultrasonographic parameters in patients with moderate CTS.

## Materials and Methods

### Patient Selection and Randomization

This study was designed as a prospective, randomized, double-blind study. It was conducted as a single-center study in the Physical Medicine and Rehabilitation Clinic of Antalya Training and Research Hospital between February 2021 and January 2022.

Before enrollment, detailed medical histories were obtained, and comprehensive physical examinations were conducted. The inclusion and exclusion criteria for the study are outlined below:

Patients aged 18-75 years, diagnosed with bilateral moderate CTS confirmed by EDx according to Padua et al’s^15^ classification criteria, and experiencing symptoms for at least 3 months were included in the study. Exclusion criteria comprised a history of prior CTS surgery or injection, presence of thenar atrophy, polyneuropathy, another upper extremity entrapment neuropathy, cervical radiculopathy, pregnancy, or being within 6 months postpartum.

Before initiating the study, all participants provided informed consent and signed a voluntary consent form. All participants were informed about the study details and provided written informed consent before enrollment. Approval was obtained from the Antalya Training and Research Hospital Clinical Research Ethics Committee (decision number 1/20, dated January 9, 2020) and the Turkish Medicines and Medical Devices Agency (document number 66175679-514.04.01-E.240680, dated October 23, 2020). The study was conducted by the principles of the Declaration of Helsinki.

### Outcome Measures

The primary outcome variables of the study were the Boston Carpal Tunnel Syndrome Questionnaire Symptom Severity Scale (BCTQ-SSS) and Functional Status Scale (BCTQ-FSS) scores. Secondary outcome measures included hand grip strength (GS), median nerve cross-sectional area (MNSA), median nerve flattening ratio (MNFR), and electrophysiological parameters.

### Clinical Evaluation

Before the injection, demographic data, including age, gender, education level, occupation, CTS stage, symptom duration, dominant hand, and history of prior injections, were recorded for all participants. Symptom severity and functional status were assessed using the BCTQ, while GS was measured using a Jamar dynamometer (Asimow Engineering, Los Angeles). Median nerve parameters, including anteroposterior (AP) diameter, transverse diameter, and MNSA, were evaluated using USG. The MNFR was calculated as the ratio of transverse to AP diameter. Nerve conduction studies of all patients were performed in the electrophysiology laboratory using Nihon Kohden Neuropack S1 MEB-9400K EMG device (Tokyo, Japan). Comparative median and ulnar nerve sensory and motor conduction velocity, distal and proximal latency, amplitudes, and ulnar nerve F responses of both upper extremities were recorded.

All parameters were assessed separately for each hand. Each participant underwent evaluations at 3 time points: before the injection, 1 month after the injection, and 6 months after the injection. Each patient was advised to use a wrist splint during the follow-up period.

Injections were administered to both hands using the same technique for the same patient. In the statistical analysis, each hand was evaluated independently.

### Interventions

A clinician with 15 years of experience in physiatry administered 1 mL (40 mg) of triamcinolone to all participants. In the first group (group 1), a LM-guided injection was performed at the level of the distal wrist crease, targeting the carpal tunnel at a 30-degree angle to the ulnar side of the palmaris longus tendon. In the second group (group 2), the injection was administered using an ulnar-sided in-plane technique under USG guidance with a 5-12 MHz linear array transducer.

In the LM-guided injection group, sonographic measurements of the median nerve were recorded using USG; however, no ultrasound images were obtained during the injection. This approach ensured that patients remained unaware of their group assignment, thereby maintaining blinding. All outcome measures were assessed by an independent researcher unaware of group allocations, ensuring a double-blind study design.

### Calculation of the Sample Size

The sample size was calculated using G*Power, Version 3.1.9.4 (Faul, Erdfelder, Buchner, & Lang; Heinrich Heine University; Düsseldorf, Germany). Based on an effect size of *d* = 0.50, *α* = 0.05, and a power of 0.80, the required sample size for each group was determined to be 64 wrists. Considering the long-term follow-up and an anticipated 30% loss to follow-up, a total of 168 wrists meeting the inclusion criteria were included, with 84 wrists per group. Randomization was performed using the closed-envelope method, assigning patients into either the LM-guided or USG injection groups.

### Statistical Analysis

Descriptive statistics are presented as frequency, percentage, mean, standard deviation, median, minimum, and maximum values. For categorical data analysis, Fisher’s Exact Test was used if more than 20% of the expected cell counts were below 5, while the Pearson Chi-square test was applied otherwise. The normality assumption was assessed using the Shapiro–Wilk Test.

For comparisons between 2 groups, the Independent Samples *t*-test was used when numerical data were normally distributed, whereas the Mann–Whitney *U*-test was applied for non-normally distributed data. Repeated-measures ANOVA (analysis of variance) was conducted to compare pre-injection, 1-month, and 6-month measurements of continuous variables across groups. The generalized estimating equation (GEE) method was used to analyze pre-injection, 1-month, and 6-month measurements of ordinal and nominal variables between groups.

The GEE analysis was performed using the PROC GEE procedure in SAS software, Version 9.4 (SAS Institute Inc.; Cary, NC, USA), while all other statistical analyses were conducted using IBM SPSS Statistics for Windows, Version 23.0 (IBM Corp.; Armonk, NY, USA). A *P* value of <.05 was considered statistically significant.

## Results

**Figure 1.**
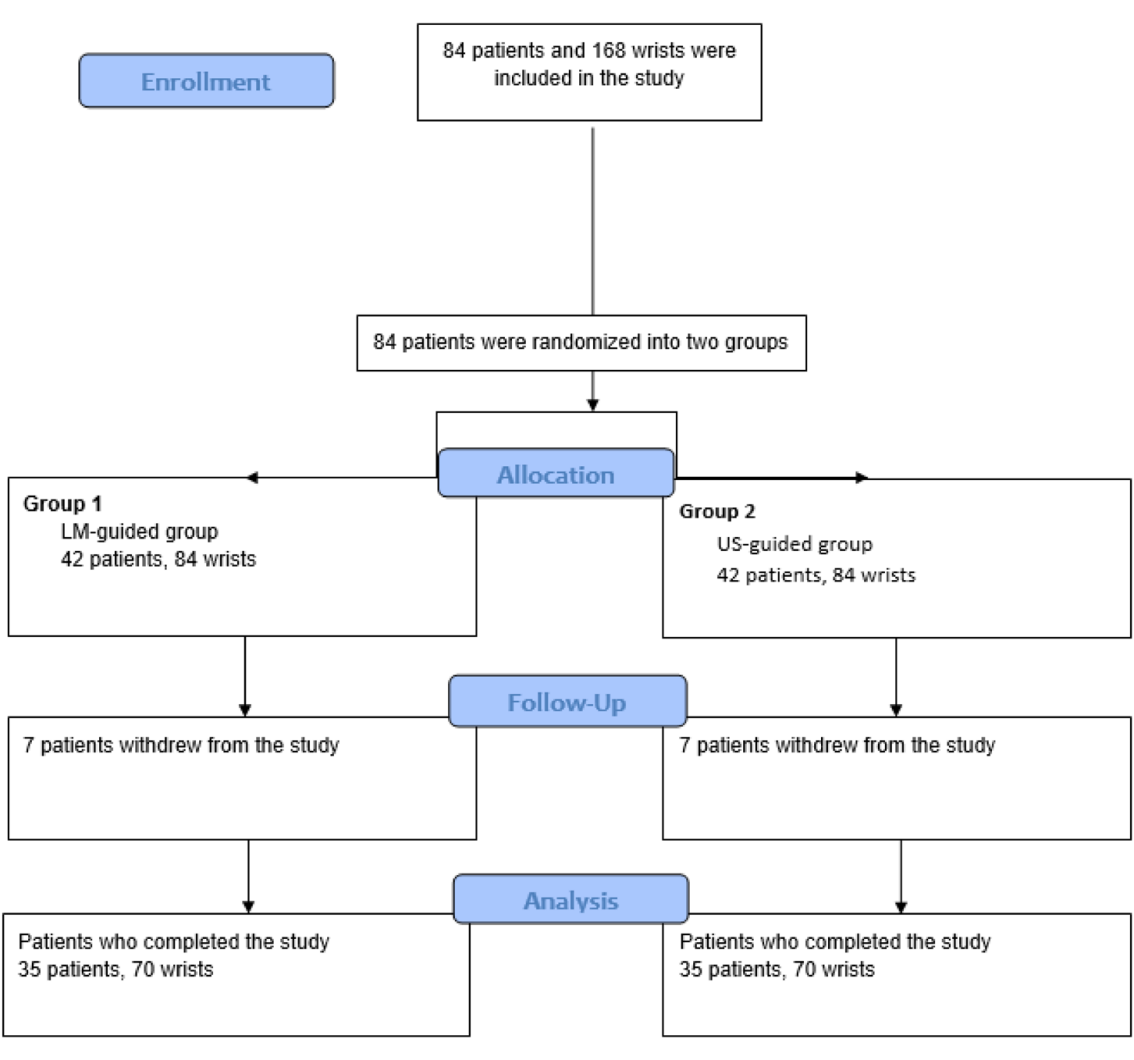
Demographics

The study included 84 participants (168 wrists), with 42 individuals in each group, all diagnosed with bilateral moderate CTS. Due to non-compliance with follow-up, 7 participants from each group were unable to complete the study. Consequently, 35 patients (70 wrists) per group completed the study (Figure 1).

No statistically significant difference was found between the 2 groups in terms of age, gender, and hand dominance, although the duration of symptoms was longer in group 2 (Table 1).

Analysis of functional and symptom severity scores in the BCTQ-SSS and BCTQ-FSS revealed no significant differences between the 2 groups. However, when baseline values were compared with the first- and sixth-month results, both groups demonstrated significant improvement. Similarly, no significant difference was found between the 2 groups regarding GS, MNSA, and MNFR parameters, yet both groups showed significant improvement at the first and sixth months compared to baseline (Table 2).

Electrophysiological parameters, including median nerve sensory and motor conduction velocities, amplitudes, and distal latencies, exhibited significant and similar improvements in both groups (Table 3). The only observed side effect was hypopigmentation, which occurred significantly more frequently in the LM-guided injection group [8 (11%) in group 1, 2(3%) in group 2, *P* = .04].

## Discussion

In this study, significant improvements were observed in the BCTQ-SSS and BCTQ-FSS scores, MNSA, MNFR, GS, and EDx parameters at 1 and 6 months post injection for both the LM-guided and USG groups. Despite these positive outcomes, no significant differences emerged between the 2 techniques at any measured time points, underscoring a parity in efficacy for the primary treatment outcomes. Importantly, while there were no severe adverse effects such as nerve damage, tendon rupture, or infections reported, minor steroid-associated skin complications like hypopigmentation were more prevalent in the LM-guided group.

The administration of local corticosteroid injections into the carpal tunnel is a well-established therapeutic approach, extensively documented for its effectiveness in reducing inflammation, alleviating edema, and decreasing mechanical compression on the median nerve. This, in turn, facilitates the healing process. These injections can be performed using either the LM-guided or the USGtechnique. Contemporary literature highlights that USG injections not only enhance therapeutic outcomes but also improve safety by reducing risks associated with blind injections, such as inaccurate steroid placement and related complications.^16^^,^^17^ Furthermore, the growing use of ultrasound in the management of musculoskeletal disorders has been transformative, offering real-time imaging that facilitates precise assessment and treatment. Its advantages, being low-cost, radiation-free, and portable, further increase its practicality and accessibility for clinicians.

The ongoing debate in the field revolves around the relative merits of USG vs. LM methods in the context of CTS. A prior meta-analysis, which included 3 randomized controlled trials, indicated that USG steroid injections were more effective in reducing symptom severity compared to LM-guided injections, although they did not significantly affect functional severity.^18^ A more recent meta-analysis incorporated a total of 8 articles. It found that individuals receiving USG injections had lower symptom severity scores on the BCTQ, a decreased risk of any complications, and a reduced likelihood of requiring surgical intervention, compared to those treated with the LM approach. All other parameters, including the FSS score, Visual Analogue Scale, and GS, as well as EDx findings, remained similar between the 2 groups.^19^ In contrast, recent studies by Farfour et al^20^ or Rathoor et al^21^ strongly advocate for the use of ultrasound guidance, highlighting its effectiveness in symptom relief and its association with lower complication rates.Although cost analysis was not performed in the study, a randomized controlled study demonstrated that ultrasound-guided injections are cost-effective, as they resulted in lower expenses for responders compared to the blind injection group. This cost reduction was found to be primarily due to fewer reinjections and fewer referrals for surgery.^16^

The findings, however, present a nuanced view, aligning with studies like those conducted by Üstün et al^22^ and Eslamian et al,^23^ which reported no significant differences between USG and LM-based methods in terms of improvement in BCTQ scores and other functional assessments. Notably, while those studies had a 12-week follow-up period, the study extended the follow-up to 6 months, further supporting the conclusion that both techniques can yield equally effective results when performed accurately over a 12-week follow-up period.

The fact that some studies have reported the superiority of USG injections while others have found comparable outcomes with LM-guided methods may be attributed to several factors. This disparity could stem from differences in study designs, the type and dosage of injectates, or the severity of CTS among patient groups. In particular, the proficiency of the clinician performing LM-guided injections might significantly influence the effectiveness of the intervention in blinded CTS injection studies.^14^ In the present study, all injections were performed by a physiatrist with 15 years of experience, and only patients with moderate CTS were included. These factors may have contributed to achieving similar efficacy in the LM-guided group.

The study extends the existing literature by examining these interventions over a 6-month period, which is notably longer than the duration considered in many comparable studies. This longer follow-up provides valuable insights into the medium-term efficacy and safety of these treatment modalities.

It is noteworthy that the only side effect observed in the study was hypopigmentation. This is a recognized complication of corticosteroid injections for CTS, occurring in approximately 6% of cases.^24^ Corticosteroids may leak into the surrounding tissue, potentially leading to atrophy of the fat tissue and skin discoloration. In the present study, the incidence of hypopigmentation was significantly lower in the USG group, likely due to the more accurate placement of the injection. This finding highlights the potential of ultrasound guidance to minimize adverse effects associated with steroid injections. The absence of complications other than hypopigmentation in either group, along with the observed clinical improvements, supports the conclusion that a corticosteroid injection is a safe and effective treatment option for CTS.

The main limitation of the study is that the follow-up period was 6 months, and only patients with moderate CTS were included. Another limitation of this study is that both hands from the same participants were included in the analysis. As data from the same individual are not entirely independent, this may have introduced a potential violation of the assumption of statistical independence. Therefore, the results should be interpreted with caution, as this factor might have slightly influenced the statistical outcomes. An important point to consider is the baseline imbalance in symptom duration between the 2 groups. The USG group had a significantly longer mean symptom duration compared with the control group. This difference may have influenced the treatment response, as longer symptom duration could be associated with more chronic tissue changes and potentially reduced responsiveness to intervention. Although randomization was performed, this imbalance might have occurred by chance. Therefore, the results should be interpreted with caution, taking this confounding factor into account. These limitations should be taken into account when evaluating the results of the study, and it should be kept in mind that generalization to all patients with CTS would not be appropriate.

## Conclusion

This study contributes to evidence suggesting that USG and LM-guided injections effectively manage moderate CTS, providing significant clinical, electrophysiological, and sonographic improvements over 6 months. Although the results demonstrate that USG may help minimize the risk of certain side effects like hypopigmentation, LM-guided injections still represent a viable and effective option, especially for experienced clinicians familiar with the anatomical nuances of the carpal tunnel who may not have ready access to ultrasound technology.

## Figures and Tables

**Table 1.** Demographics

		**Group 1 (n = 70)**	**Group 2 (n = 70)**	***P***
Age, years		49.6 ± 10.6	47.94 ± 9.33	.26
Sex, n (%)	Female	58 (82.9)	64 (91.4)	.13
	Male	12 (17.1)	6 (8.6)	
Duration of symptoms, months		36.1 ± 28.9	51.3 ± 40.0	.049
Dominant hand, n (%)	Right	62 (88.6)	66 (94.3)	.23
	Left	8 (11.4)	4 (5.7)	

**Table 2.** Functional and Symptom Severity Scores Results

	**Groups**	**Baseline**	**First Month**	**Sixth Month**	**Group Effect**	**Group × Time**	**Time Effect**	***P1***	***P2***
BCTQ-SSS	Group 1	3.08 ± 0.77	1.85 ± 0.68	1.94 ± 0.91	*F* = 0.269 *P* = .605	*F* = 0.010 *P* = .989	***F* = 169.156** ***P*** ** < .0001**	**<.0001**	**<.0001**
	Group 2	3.02 ± 0.83	1.81 ± 0.73	1.89 ± 0.70					
BCTQ-FSS	Group 1	3.16 ± 1.00	2.05 ± 0.91	2.02 ± 1.11	*F* = 1.055 *P* = .306	*F* = 0.128 *P* = .872	***F* = 107.711** ***P*** ** < .0001**	**<.0001**	**<.0001**
	Group 2	3.24 ± 0.74	2.19 ± 0.88	2.19 ± 0.95					
GS (kg)	Group 1	21.12 ± 8.54	24.61 ± 9.48	24.05 ± 10.03	*F* = 1.100 *P* = .296	*F* = 0.359 *P* = .690	***F* = 32.727** ***P*** ** < .0001 **	**<.0001**	**<.0001**
	Group 2	19.97 ± 7.65	22.77 ± 7.97	22.76 ± 7.38					
MNSA (cm^2^)	Group 1	0.136 ± 0.041	0.117 ± 0.035	0.122 ± 0.038	*F* = 0.022 *P* = .966	***F* = 3.94** ***P*** ** = .024**	***F* = 37.81** ***P*** ** < .0001**	**<.0001**	**<.0001**
	Group 2	0.139 ± 0.039	0.122 ± 0.036	0.114 ± 0.030					
MNFR	Group 1	3.42 ± 0.95	3.56 ± 0.83	3.66 ± 0.80	*F* = 0.018 *P* = .895	*F* = 0.228 *P* = .796	***F* = 7.22** ***P*** ** = .001**	.075	**.001**
	Group 2	3.38 ± 0.75	3.58 ± 0.82	3.72 ± 1.04					

BTCQ FSS, Boston Carpal Tunnel Syndrome Questionnaire Functional Status Scale; BTCQ SSS, Boston Carpal Tunnel Syndrome Questionnaire Symptom Severity Scale; GS, grip strength; MNFR, median nerve flattening ratio; MNSA, median nerve sectional area.

*P*1: *P* value after baseline and first-month comparison, *P*2: *P* value after baseline and sixth-month comparison.

**Table 3.** Electrophysiological Results

	Groups	Baseline	First Month	Sixth Month	Group Effect	Group × Time	Time Effect	*P1*	*P2*
MDL (ms)	Group 1	4.82 ± 0.87	4.17 ± 0.72	3.96 ± 0.57	*F* = 1.34 *P* = .249	*F* = 3.98 **P = .029**	*F* = 173.781 ***P*** ** < .0001**	**<.0001**	**<.0001**
	Group 2	4.60 ± 0.70	4.02 ± 0.54	3.97 ± 0.56					
CMAP (µV)	Group 1	11.75 ± 3.55	12.64 ± 3.57	13.15 ± 3.04	*F* = 2.394 *P* = .124	*F* = 1.741 *P* = .189	*F* = 18.18 ***P*** ** < .0001**	**<.0001**	**<.0001**
	Group 2	12.26 ± 3.71	14.03 ± 4.03	13.70 ± 3.70					
MNCV (m/s)	Group 1	54.88 ± 3.47	55.55 ± 3.47	55.17 ± 3.00	*F* = 12.291 ***P*** ** = .001**	*F* = 1.882 *P* = .154	*F* = 2.461 *P* = .087	.493	.091
	Group 2	56.40 ± 3.16	56.70 ± 4.22	57.59 ± 4.48					
SDL (ms)	Group 1	3.96 ± 0.68	3.52 ± 0.58	3.33 ± 0.42	*F* = 0.515 *P* = .474	*F* = 1.322 *P* = .266	*F* = 96.29 ***P*** ** < .0001**	**<.0001**	**<.0001**
	Group 2	3.85 ± 0.75	3.43 ± 0.47	3.35 ± 0.58					
SNAP (µV)	Group 1	19.50 ± 9.04	23.03 ± 8.12	24.31 ± 7.33	*F* = 5.082 ***P*** ** = .026**	*F* = 1.291 *P* = .275	*F* = 42.155 ***P*** ** < .0001**	**<.0001**	**<.0001**
	Group 2	21.45 ± 9.29	26.20 ± 10.55	28.36 ± 10.34					
SNCV (m/s)	Group 1	34.05 ± 6.33	38.99 ± 7.32	40.14 ± 5.02	*F* = 0.703 *P* = .403	*F* = 0.378 *P* = .685	*F* = 114.49 ***P*** ** < .0001**	**<.0001**	**<.0001**
	Group 2	33.67 ± 5.33	37.89 ± 5.02	39.38 ± 6.32					

CMAP, compound muscle action potential amplitude; MDL, motor distal latency; MNCV, motor nerve conduction velocity; SD, sensory distal latency; SNAP, sensory nerve action potential amplitude; SNCV, sensory nerve conduction velocity.

*P*1, *P* value after baseline and first-month comparison; *P*2, P value after baseline and sixth-month comparison.

## Data Availability

The data that support the findings of this study are available from the corresponding author upon reasonable request.
